# Visualization Monitoring and Safety Evaluation of Turnout Wheel–Rail Forces Based on BIM for Sustainable Railway Management

**DOI:** 10.3390/s25144294

**Published:** 2025-07-10

**Authors:** Xinyi Dong, Yuelei He, Hongyao Lu

**Affiliations:** 1 College of Urban Rail Transportation, Shanghai University of Engineering Science, Shanghai 201600, China; m405123403@sues.edu.cn (X.D.); hyldoc@163.com (Y.H.); 2 Shanghai Key Laboratory of Structural Durability and System Safety of Rail Transit, Tongji University, Shanghai 201800, China

**Keywords:** high-speed railway, Building Information Modeling, turnout wheel–rail force monitoring, 4D construction simulation, data analysis, sustainable operation and maintenance

## Abstract

With China’s high-speed rail network undergoing rapid expansion, turnouts constitute critical elements whose safety and stability are essential to railway operation. At present, the efficiency of wheel–rail force safety monitoring conducted in the small hours reserved for the construction and maintenance of operating lines without marking train operation lines is relatively low. To enhance the efficiency of turnout safety monitoring, in this study, a three-dimensional BIM model of the No. 42 turnout was established and a corresponding wheel–rail force monitoring scheme was devised. Collision detection for monitoring equipment placement and construction process simulation was conducted using Navisworks, such that the rationality of cable routing and the precision of construction sequence alignment were improved. A train wheel–rail force analysis program was developed in MATLAB R2022b to perform signal filtering, and static calibration was applied to calculate key safety evaluation indices—namely, the coefficient of derailment and the rate of wheel load reduction—which were subsequently analyzed. The safety of the No. 42 turnout and the effectiveness of the proposed monitoring scheme were validated, theoretical support was provided for train operational safety and turnout maintenance, and technical guidance was offered for whole-life-cycle management and green, sustainable development of railway infrastructure.

## 1. Introduction

Under the guidance of the national strategy for establishing a transportation powerhouse in the new era, the scale of high-speed railway construction in China has continuously expanded, and its technological system has been iteratively upgraded. A collaborative framework for the intelligent monitoring of infrastructure structural health and sustainable lifecycle management has been established as a core proposition for ensuring the safe and efficient operation of the high-speed rail network and for achieving high-quality development of the industry [1,2].

As the critical components that guide trains in smooth and safe track transitions, railway turnouts are endowed with technical systems integrated from multiple disciplinary fields and have been regarded as the most complex and challenging elements of rail track structures. They have also been identified as the weak link of the track and as key facilities restricting train passage speeds, and, owing to their technical complexity, have been recognized as the core technological bottleneck constraining the safe and efficient operation of railway systems [3]. The safety, stability and operational efficiency of the railway system are directly influenced by the operational performance of the turnouts [4].

The contact forces generated by interactions at the wheel–rail interface are regarded as critical factors influencing the dynamic performance, safety, and smooth operation of railway vehicles [5]. When high-speed and heavy-haul trains traverse turnouts, variations in the wheel–rail forces are characterized by a high level of complexity, and the turnout structure itself is defined by significant geometric intricacy, which can result in wear, fatigue damage, and even train derailment or overturning [6,7]. The accurate monitoring and scientific analysis of wheel–rail forces have been identified as essential for enhancing turnout operational safety and reducing maintenance costs.

Under the prevailing requirement that equipment maintenance be conducted during line possession windows, the formulation and implementation of traditional high-speed railway wheel–rail force monitoring schemes have been based predominantly on engineering monitoring experience and construction drawings, with sensor numbering and deployment locations typically presented via two-dimensional diagrams and textual descriptions. Although such methods can satisfy turnout area force-monitoring demands to a certain extent, low efficiency, significant construction interference, and high measurement error rates have been encountered [8,9]. Consequently, not only has turnout system safety been constrained, but increased energy consumption, material resource redundancy, and labor resource misallocation have also been incurred, giving rise to marked contradictions with the strategic objectives of a green, low-carbon transformation in the railway industry. Therefore, the construction of a multidimensional integrated information management system has been recognized as an urgent requirement. To achieve sustainability enhancements across the design, manufacturing, construction, installation, and operation-and-maintenance phases, collaborative innovation among stakeholders from diverse professional domains must be conducted throughout the entire lifecycle [10].

With Building Information Modeling (BIM) technology regarded as a key technique for digital-twin construction, rapid development has been witnessed in the infrastructure domain [11]. Visualization-based layout, construction simulation, and collaborative lifecycle management, all founded on three-dimensional digital models, have been progressively adopted as vital approaches for enhancing green construction and intelligent operation-and-maintenance capabilities [12]. BIM technology enables three-dimensional modeling and semantic representation of turnout components, providing a reliable framework for real-time condition monitoring, sensor deployment, and maintenance decision-making. Recent studies have demonstrated the effectiveness of integrating BIM with accelerometer data and machine learning algorithms to automatically detect anomalies such as tight and wide gauges [13,14] and track geometry deformations [15], thereby improving the safety and cost-efficiency of railway maintenance strategies. The coupling of BIM with Internet of Things (IoT) and digital twin technologies has also allowed for real-time structural health monitoring (SHM) of railway bridges [16], predictive maintenance scheduling [17], and lifecycle-based climate resilience assessment [18].

Furthermore, BIM has been successfully applied in various domains of infrastructure monitoring, including long-span bridge deformation control [19], pipeline corrosion detection [20], and hazard visualization through sensor-BIM integration. These applications highlight BIM’s versatility in managing complex data streams, optimizing sensor placements, and facilitating collaborative workflows across disciplines. Especially in railway infrastructure, BIM-based SHM has been extended to cover tasks such as load monitoring, fatigue analysis, and maintenance prioritization using higher-dimensional data models and intelligent analytics [21,22].

Fine-scale modeling of complex structures has been enabled by BIM technology to represent real-world entities, thereby facilitating more efficient and reliable communication among stakeholders and enhancing user productivity. The production of visualized handover documentation is carried out as a key engineering practice within BIM applications; through images, animations, and similar media, the various stages and critical considerations of the construction process are intuitively presented, resulting in improved construction efficiency and quality and reductions in rework, misassembly, and waste [23].

The No. 42 turnout was selected as the research object for this study to systematically investigate the scientific layout of monitoring devices and visualization-guided techniques based on Building Information Modeling (BIM) technology. A dynamic process simulation was generated to direct actual construction operations, and the validity and feasibility of the proposed scheme were verified through on-site measurements and data analysis, thereby ensuring an orderly and efficient implementation of the wheel–rail force monitoring system for turnouts. As a result of this study, construction conditions, organizational arrangements, and specific key technical measures for wheel–rail force monitoring can be more clearly defined by technical personnel; the entire monitoring process and its critical locations can be systematically mastered; and work scope, operational procedures, and safety precautions can be fully understood. Not only are train operation safety and turnout maintenance efficiency enhanced, but theoretical foundations and engineering references for realizing intelligent operation and maintenance, green construction, and sustainable management of railway turnouts are also provided.

## 2. Establishment of the Turnout Wheel–Rail Force Monitoring Model and Monitoring Layout

### 2.1. Establishment of Turnout and Monitoring Equipment Models

The No. 42 single turnout (hereinafter referred to as the No. 42 turnout) is a high-speed railway turnout independently designed and developed in China, with a frog angle (the angle formed by the turnout center) of 1°21′50.13″. Its straight-through allowable speed is 350 km/h, and the lateral allowable speed is 160 km/h. It is the turnout with the largest number and the highest lateral allowable speed that has been trial-produced and laid in China so far. Moreover, its planar alignment still follows the French “circle + curve” alignment. Due to the cotangent value of its frog angle being 42, it is named the No. 42 turnout.

The three-dimensional model of the turnout was established on the basis of BIM technology as the foundation for wheel–rail force monitoring and was carried through the design, construction, and operation/management stages, thereby providing novel solutions for turnout wheel–rail force monitoring [24]. In this study, the No. 42 turnout was taken as an example, and, in accordance with the design drawings and construction standards, BIM software (Revit 2020) was employed to transform the two-dimensional turnout schematics into a three-dimensional digital model. The model encompassed not only the basic geometric configuration of the turnout but also the material properties and structural dimensions of its components, together with their connection relationships to a ballastless double-block track system, thus achieving multidimensional information integration from geometric form through material attributes and construction processes to operation management [25,26].

During the turnout modeling process, particular attention was paid to the geometric configurations of key locations—namely, the parting point, frog, and switch rails—to facilitate subsequent integration with monitoring data. Based on the characteristics of the monitoring object, the frog area of the No. 42 turnout was subdivided into the following key subcomponents: switch rails, fasteners, point machines, and closure rails, as well as cast-in-place concrete sleepers, track slabs, bases, and bridge elements. Corresponding parametric families were respectively defined for each subcomponent and were assembled to form the No. 42 turnout model.

**Figure 1:**
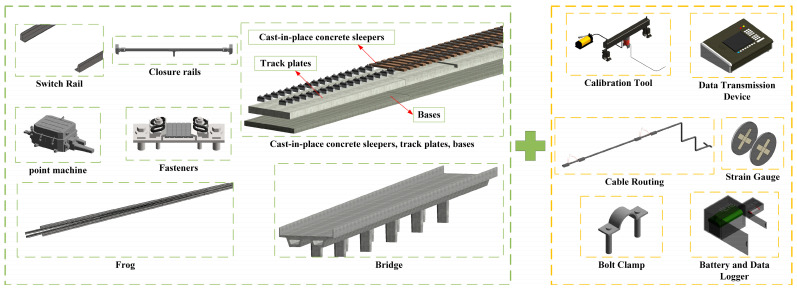
Model of track structure and monitoring equipment.

A monitoring equipment model was established as a foundation for optimizing sensor-deployment schemes and simulating operational workflows. In this study, the continuous ground-based method was employed for wheel–rail force testing, and strain gauges, data acquisition units, computation terminals, and batteries were selected for wheel–rail force data collection. To further support the wheel–rail force monitoring system, a family of parametric models representing strain gauges, data acquisition units, computation terminals, and batteries was constructed, as illustrated in Figure 1. Protective fixtures for the acquisition units and batteries were designed to mitigate environmental influences and to ensure continuity of the monitoring process. This modular digital modeling approach not only facilitates subsequent maintenance and replacement but also provides data support for full lifecycle equipment management, constituting a crucial element in achieving sustainable infrastructure operation and maintenance. The arrangement of monitoring devices was optimized according to turnout structural features and monitoring demands to ensure that force variations within the turnout were comprehensively captured.

### 2.2. Monitoring Content and Selection of Monitoring Locations

**Figure 2:**
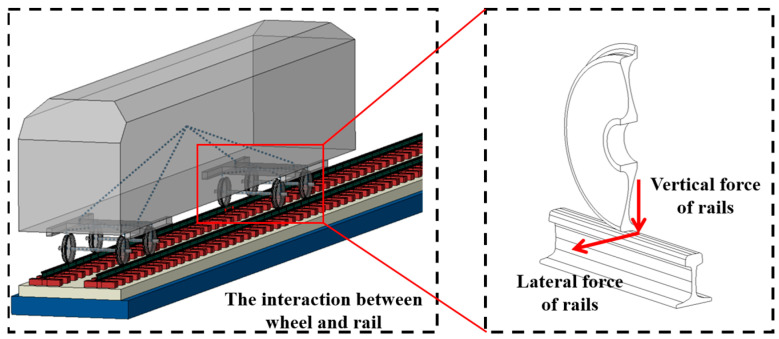
The interaction between the wheel and rail.

During train operation, the interaction between the wheel and the rail generates lateral and vertical contact forces, which are fundamental to determining the vehicle’s dynamic behavior and operational safety, as shown in Figure 2. At key geometric discontinuities within the turnout structure, such as the switch rail entry, sudden changes in track geometry and gauge can lead to abrupt variations in the wheel–rail forces. These irregularities can result in relative displacement between wheel and rail, significantly increasing the risk of wheel climb or derailment. Therefore, monitoring wheel–rail interaction at these specific locations provides valuable insights into the operational condition of the turnout. By acquiring force data at these points and computing safety evaluation indicators, such as the coefficient of derailment and the rate of wheel load reduction, a comprehensive understanding of the turnout’s dynamic performance can be achieved, offering theoretical support for both structural optimization and derailment risk mitigation.

**Figure 3:**
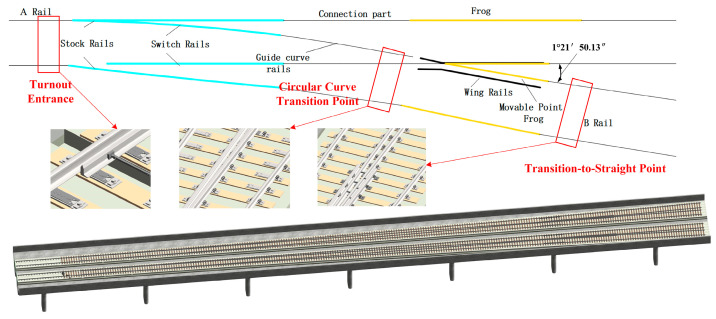
Distribution of measurement point positions for the No. 42 turnout.

Significant differences in wheel–rail force variations are observed across different segments of a turnout, making rational arrangement of monitoring points essential. In order to ensure that the monitoring system is able to reflect the force variations of the turnout comprehensively and to promptly identify potential safety hazards, key locations of the turnout have been selected as focus monitoring areas [27]. The entrance region of the turnout constitutes the initial area of train entry, in which the switch rail and stock rail remain in contact, and a pronounced stiffness discontinuity leads to significant vibrations and wheel–rail force impacts, thereby increasing the risk of wheelset uplift. The transition point between the circular curve and the easement curve represents a segment wherein complex interactions between centrifugal force and lateral wheel–rail force occur, often resulting in uneven force distribution and elevated friction. The transition from the easement curve to the straight track, being the point at which the train exits the curve onto the tangent track, can reflect a dynamic recovery state following curve negotiation [28,29,30,31]. Accordingly, the above three monitoring points were selected for equipment deployment and cable routing. The measurement point positions are illustrated alongside a schematic of the No. 42 turnout model in Figure 3.

Utilizing BIM technology, sensors were accurately positioned at key locations within the three-dimensional turnout model, and parameters such as orientation and embedment depth were ensured to be appropriate. Consistency between the device layout and the actual turnout structure was achieved, and potential installation issues were identified in advance by means of the BIM model.

## 3. Design and Optimization of the Wheel–Rail Force Monitoring Scheme

### 3.1. Design of the Wheel–Rail Force Monitoring Scheme

The design of a wheel–rail force monitoring scheme is a complex and multi-layered process encompassing several critical components, including the layout of sensing elements, cable routing methods, construction of data acquisition and transmission systems, installation techniques for monitoring equipment, and optimization of operational procedures [32]. Traditional methods are typically “experience-driven” and often overlook resource efficiency and sustainability of operations and maintenance, leading to problems such as equipment redundancy, disorganized cable arrangements, and construction conflicts. To ensure the efficient operation of the system and the accuracy of the collected data, all design components must be coordinated and compatible with one another in a holistic manner [33].

The layout of sensing elements must be determined by comprehensively considering turnout geometry, train operational characteristics, and sensing principles so as to ensure the accuracy and completeness of force measurements. Cable routing must account for the complexity of the site environment while minimizing external interference and equipment failures.

Regarding data acquisition and transmission, the monitoring devices must be capable of real-time data collection, transmission, and storage, and must possess efficient data processing capabilities. To ensure data timeliness and accuracy, the selection of monitoring equipment must align with existing communication protocols and network architectures, guaranteeing stable signal transmission and efficient data storage. Additionally, the design of the data analysis and processing software is of critical importance.

Installation locations for equipment must be determined based on the actual structural characteristics of the turnout and track, ensuring maximum interaction between monitoring devices and train operation, while also preventing system malfunctions caused by improper installation. The installation method must be highly adaptable to meet the varying demands of different construction stages.

**Figure 4:**
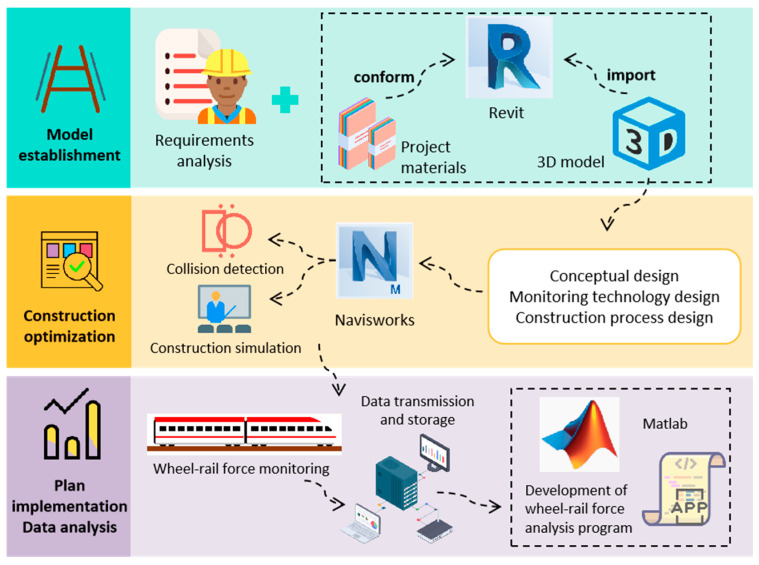
Flowchart of the wheel–rail force monitoring scheme.

The optimization of operational procedures constitutes a key component of the entire monitoring scheme design. Through rational scheduling, time wasted during device installation and commissioning can be minimized, thereby improving construction efficiency. Moreover, optimized procedures help ensure timely task completion and significantly reduce safety risks. The overall design process for the turnout wheel–rail force monitoring scheme is illustrated in Figure 4, which encompasses the entire cycle from model creation, through scheme design, simulation, and optimization, to final implementation.

### 3.2. Construction Simulation and Visualization

For a long time, construction conflicts and safety issues have significantly constrained improvements in construction quality and efficiency. These challenges have primarily arisen due to unskilled labor, insufficient safety awareness, inadequate enforcement of safety regulations, and a lack of advanced and practical safety technologies and management tools, making it difficult to accurately analyze dynamic construction processes and their safety implications. To ensure efficient interoperability between software platforms, Navisworks, a product developed by Autodesk, was selected for construction simulation. Navisworks is equipped with powerful model clash detection and construction process simulation capabilities, along with integrated time-based (4D) management functions.

Through clash detection, conflict reports can be automatically generated to identify and correct potential design issues in a timely manner. This allows construction personnel to detect possible interferences that could impact progress or structural integrity before actual implementation. By importing construction scheduling data, a dynamic simulation model was created by linking the project model with its timeline. Multiple construction schemes were simulated and validated during the design phase to help designers identify irrational procedures in advance and avoid rework caused by poor sequencing.

During the actual construction phase, comparisons between planned and actual schedules enabled real-time adjustments to the construction plan, ensuring on-time project delivery. In this way, dynamic tracking and management of the construction schedule were achieved, providing scientific support for project management [34].

#### 3.2.1. Sensor Placement Clash Detection

As steel reinforcement is embedded within the track bed slab, improper selection of drilling locations during the installation of monitoring equipment may expose the reinforcement in certain areas. In this study, the reinforcement arrangement was first obtained from the design drawings and then verified in situ using a rebar detector to account for any deviations from the as-built conditions. If rebar lacks adequate concrete coverage and protection, it becomes highly susceptible to environmental corrosion, which causes volumetric expansion. This, in turn, can induce cracks in the surrounding concrete, weaken the overall structural integrity of the track bed, reduce its durability and service life, and ultimately compromise the safety and stability of the railway infrastructure. Therefore, the drilling locations for monitoring devices were precisely analyzed in the BIM model and cross-checked against the rebar detector-confirmed reinforcement layout to account for any deviations from the as-built conditions and avoid conflict with critical reinforcement zones, and to ensure that the structural integrity and safety of the track bed were maximally preserved. To guarantee the scientific installation of stress sensors and their auxiliary components, the “Clash Detective” tool in Navisworks was employed to identify potential collisions between turnout components and embedded reinforcement. During the clash detection process, key parameters such as detection precision, clash range, and allowable tolerance were configured. For this study, a tolerance of 1–2 mm was set to ensure sufficient detection accuracy. The bolt clamp and the embedded steel reinforcement within the turnout were designated the primary detection objects, while non-critical clashes below the defined tolerance were ignored.

**Figure 5:**
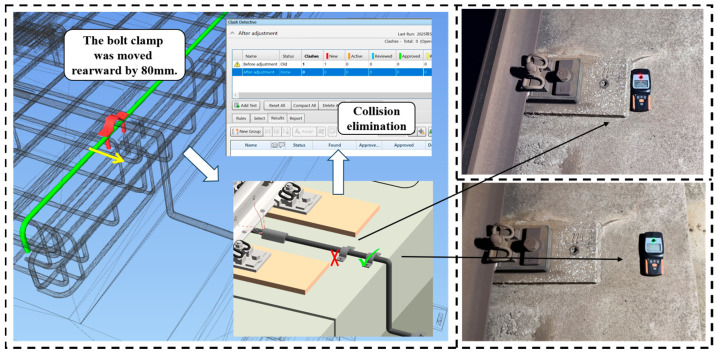
Clash detection between bolt clamp and reinforcement.

The detection process revealed reinforcement conflicts in the initial installation zone, indicating a need to adjust the bolt clamp position. After repositioning the clamp 80 mm rearward and re-running clash detection, interference with the rebar was eliminated. Thus, this adjusted drilling location was identified as the optimal solution for cable installation, as illustrated in Figure 5.

#### 3.2.2. Monitoring Process Simulation

**Figure 6:**
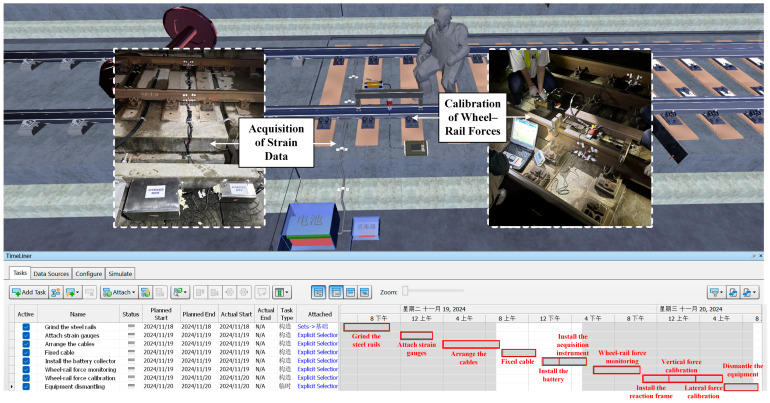
Monitoring process simulation.

To further enhance the feasibility and implementation efficiency of the monitoring scheme, this study utilized Navisworks to conduct a detailed simulation of the entire monitoring process. As shown in Figure 6, the “Timeliner” module was used to integrate key elements—including the installation sequence of monitoring equipment, cable routing paths, strain gauge placement strategy, and wheel–rail force calibration procedures—into the construction schedule framework. By simulating the construction process, the working status of each sensor, timing of data acquisition, and scheduling of force calibration were all predicted. This digital pre-execution significantly improved the reliability during the debugging phase of the monitoring system and ensured the timeliness of data collection.

A dynamic workflow model was developed using the “Animator” module, which enables visual comparison between actual and planned progress, allowing for real-time corrections of process deviations and optimization of resource allocation. This not only reduces the cost of rework due to schedule delays but also enhances the precision of construction management.

**Figure 7:**
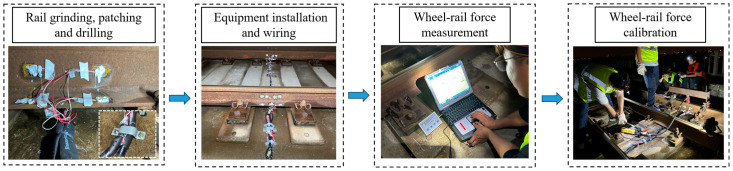
The procedure for wheel–rail force monitoring.

The specific procedure for wheel–rail force monitoring is illustrated in Figure 7 and can be described as follows:

1. Surface Preparation:

The testing process begins with grinding the steel rails to ensure a clean and uniform surface for strain gauge attachment. This step helps eliminate rust, unevenness, and other surface contaminants that could affect measurement accuracy.

2. Sensor Installation:

After surface preparation, strain gauges are attached at predetermined locations on the rails. This step requires precise positioning to capture both vertical and lateral forces accurately.

3. Cable Arrangement and Fixing:

The signal cables from the strain gauges are neatly arranged and fixed along the rail structure to minimize signal interference and avoid mechanical damage during train passage or calibration activities.

4. Power and Acquisition Unit Setup:

The battery pack and data acquisition instruments are installed in protective boxes beside the track. These units serve to power the sensors and collect real-time signal data during train operations and calibration loading.

5. Wheel–Rail Force Monitoring:

With the full monitoring system in place, real-time wheel–rail force data are recorded during train passage. This phase captures the operational loading conditions, including dynamic vertical and lateral forces.

6. Static Force Calibration:

Static force calibration is carried out by installing a reaction frame over the targeted rail section and applying known vertical and lateral loads using calibrated jacks or mechanical loading devices. The corresponding strain responses are then recorded to establish an accurate calibration relationship between the raw electrical signals collected by the strain gauges and the actual magnitudes of wheel–rail forces.

7. System Dismantling:

Upon completion of the monitoring and calibration tasks, all equipment is carefully dismantled, including sensors, cables, acquisition units, and power supplies. This ensures the track returns to its original operational state without residual equipment.

In addition, the platform’s integrated 3D walkthrough functionality offers an immersive review tool for on-site personnel, supporting free perspective switching and zoom-in inspection of model details. Construction teams can conduct multiple rounds of virtual pre-acceptance inspections to identify potential issues such as equipment clashes or insufficient operating space in advance, significantly reducing the likelihood of on-site rework. This hybrid virtual/physical validation approach enables effective control over the construction quality of the wheel–rail force monitoring system and establishes a high-precision digital benchmark for future operations and maintenance, thereby enhancing the system’s capability for full lifecycle monitoring of the turnout.

## 4. Train Wheel–Rail Force Analysis Program: Development and Application Conclusions

Compared to static calculations, the interaction forces between wheels and rails during train operation are significantly more complex. Evaluating train operation using dynamic indicators allows for a more realistic and comprehensive assessment of the turnout section’s operational performance. During train movement, wheel–rail force is a critical parameter for assessing operational safety and vehicle dynamic performance [35]. To prevent safety incidents such as overturning or derailment, real-time monitoring and analysis of wheel–rail force are essential [36].

In terms of data acquisition, this study adopts a ground-based measurement approach to capture both lateral and vertical wheel–rail forces. Although these measurements provide a fundamental representation of force behavior, the raw signal data are subject to external noise and interference. Therefore, filtering and static calibration are required before the data can be reliably used for analysis.

Based on the MATLAB platform, this study developed a dedicated analysis and application program for train wheel–rail forces in turnout zones. The system performs signal filtering, static calibration, and dynamic analysis to accurately calculate wheel–rail forces. It also derives safety-related indices such as derailment coefficient and the rate of wheel load reduction, thereby enabling a comprehensive safety evaluation of train passage through the turnout.

### 4.1. Data Acquisition, Filtering, and Computation

When the train traverses the measurement points, the external forces exerted by the wheel–rail interface induce minute deformations in the rail, which are in turn transferred to the strain gauges affixed to the rail. These strain gauges convert the mechanical deformation into electrical signals, which are received and recorded by the data-acquisition units. As various sources of interference affect the signal collection process, the raw signals typically contain noise and must be subjected to filtering prior to analysis [37].

Given the existence of a linear or nonlinear relationship between the measured strain signals and wheel–rail forces, static calibration experiments were conducted on the test segment to establish the calibration function linking strain to wheel–rail force, thereby enabling the conversion of raw signal data into accurate force measurements. By applying mathematical modeling, regression analysis, and numerical optimization methods such as the least-squares approach, the filtered strain signals were accurately transformed into wheel–rail force data [38].

The processed wheel–rail force data not only provide insight into the dynamic load conditions during train operation but also enable the calculation of safety evaluation indices, such as the coefficient of derailment and the rate of wheel load reduction, to assess the train’s running state through the turnout [39]. The coefficient of derailment, defined as the ratio of lateral to vertical wheel–rail force, reflects the relative overturning moment and the tendency for derailment, while the rate of wheel load reduction, defined as the ratio of actual to theoretical wheel load, indicates variations in axle loading during operation. Both parameters are critical for safety assessment according to the Code for “*Dynamic Performance Evaluation and Test Appraisal of Railway Vehicles*” (GB/T 5599-2019) [40].

The coefficient of derailment is specified as

Q/P ≦ 0.8(1)

where Q denotes the lateral wheel–rail force and P represents the vertical wheel–rail force. This ratio reflects the tendency of the wheel to climb over the rail, with higher values indicating an increased risk of derailment.

The rate of wheel load reduction is specified as

(2) $$\Delta P/\bar{P}≦0.65$$

where ΔP is the reduction in vertical wheel–rail force and $\bar{P}$ is the average vertical wheel–rail force. This parameter quantifies the degree of wheel unloading during vehicle passage, which is critical for evaluating wheel–rail contact stability.

**Figure 8:**
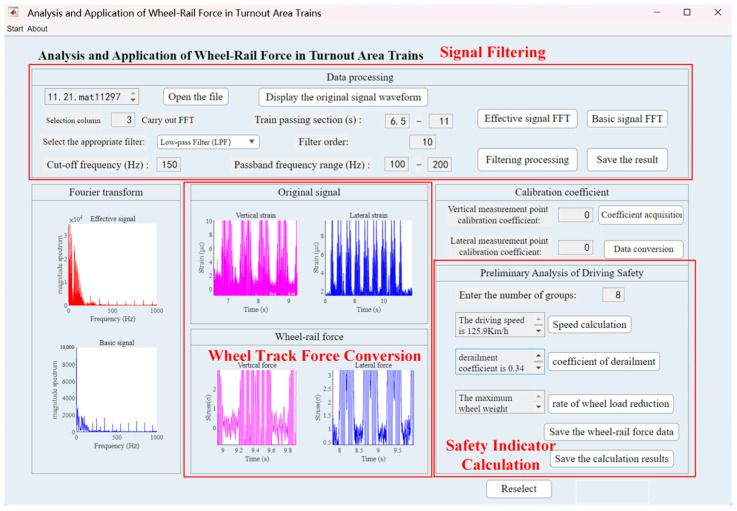
Program interface.

The MATLAB-based analysis and application program operates independently of the BIM environment. After data collection, sensor data and calibration coefficients are exported as CSV files and serve as static inputs to the MATLAB scripts, which run in single-execution mode—processing the acquired sensor readings on demand. This program is based on the wheel–rail force data obtained through static calibration and incorporates train structural characteristics, speed, and operating conditions. Numerical models are employed to compute the coefficient of derailment and the rate of wheel load reduction. The resulting safety indicators can be used to monitor and evaluate risks during train passage through the turnout. No automated runtime link exists between the BIM model and the safety evaluation module; however, all export formats and processing steps are documented in detail to ensure reproducibility. The program’s user interface is shown in Figure 8.

### 4.2. Data Analysis and Safety Evaluation

Wheel–rail force data collected at three critical locations—the turnout entrance, the circular-to-easement transition, and the easement-to-tangent transition—were further analyzed to compute the coefficient of derailment and the rate of wheel load reduction, which were then subjected to comprehensive evaluation. By comparing the coefficient of derailment and the rate of wheel load reduction across these measurement points, the safety performance of the turnout under varying operational conditions could be assessed.

**Figure 9:**
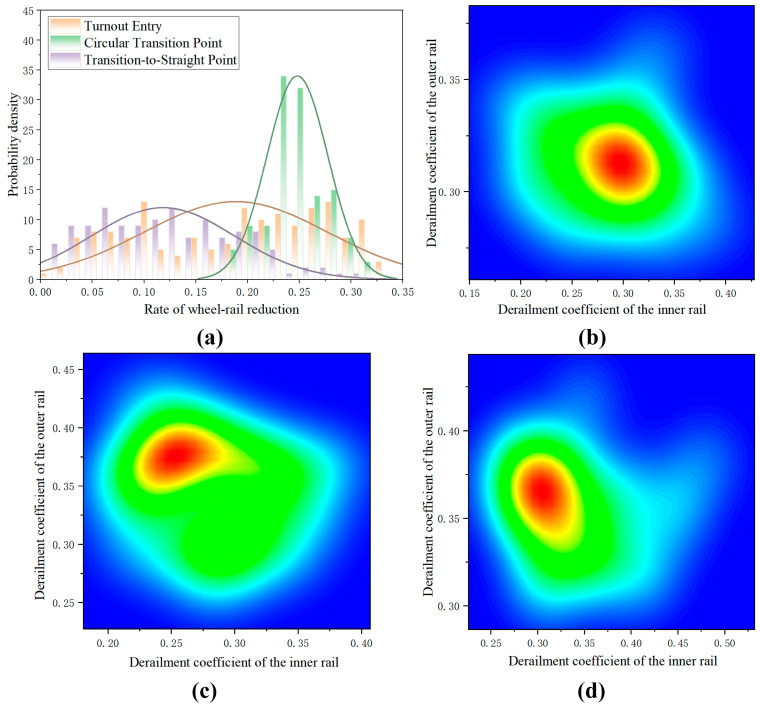
(**a**) Distribution of the rate of wheel load reduction at different zones of the turnout; (**b**) Distribution of the coefficient of derailment at the turnout entry; (**c**) Distribution of the coefficient of derailment at the circular transition point; (**d**) Distribution of the coefficient of derailment at the transition-to-straight point. (In subfigures (**b**–**d**), a color gradient transitions from blue to red. Blue indicates low data frequency/small probability, while red represents high data frequency/large probability).

In Figure 9, the distribution of the rate of wheel load reduction at the turnout entrance, the circular-to-easement transition, and the easement-to-tangent transition is shown, and a generally normal distribution is observed. Compared with the turnout entrance and the easement-to-tangent transition, the circular-to-easement transition exhibited the highest mean rate of wheel load reduction, approximately 0.25, indicating that an elevated risk of wheel uplift and derailment is posed by wheel load reduction at this location. Among the three measurement points, the smallest rate of wheel load reduction, approximately 0.18, was recorded at the easement-to-tangent transition.

As depicted in Figure 9b–d, at the turnout entrance the inner and outer coefficients of derailment were of similar magnitude, whereas at the other two points the outer coefficient of derailment was found to exceed the inner coefficient, likely due to the increased centrifugal force experienced during curve negotiation. At the circular-to-easement transition, the inner coefficient of derailment was concentrated between 0.24 and 0.27, while the outer coefficient of derailment was concentrated between 0.36 and 0.38.

**Figure 10:**
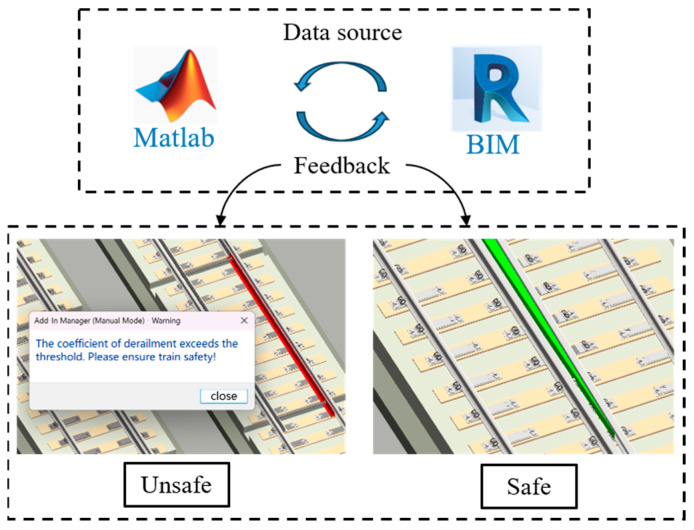
Status alarm.

Although the safety evaluation calculations are performed in MATLAB, the resulting derailment coefficients and wheel load reduction rates are imported into the BIM model for three-dimensional visualization. These indices are mapped onto the 3D turnout. When a derailment coefficient or wheel load reduction rate exceeds the safety threshold, the affected region is automatically highlighted in red, providing visual alerts, as shown in Figure 10. This enables stakeholders to intuitively identify high-risk areas, thereby supporting more informed decision-making for maintenance planning and design optimization.

Nevertheless, the coefficient of derailment and the rate of wheel load reduction at all three measurement points remained below the safety limits specified by the national standard, indicating that the train’s operating condition within the turnout zone was satisfactory. It is recommended that subsequent investigations focus on the wheel–rail force conditions at the circular-to-easement and easement-to-tangent transitions, and that structural designs be optimized or buffer measures incorporated to reduce impact forces and enhance turnout safety.

By combining parameters such as train speed, coefficient of derailment, and rate of wheel load reduction, a comprehensive evaluation of train operational safety can be provided by the program, and theoretical foundations for turnout maintenance and design optimization can be furnished. This approach not only improves the analytical accuracy of the wheel–rail force monitoring system but also establishes a reliable tool for safety assessment and management within the railway industry.

## 5. Conclusions

To address the complex requirements of wheel–rail force monitoring in high-speed railway turnouts, a comprehensive, BIM-based full-process solution—comprising structural modeling, sensor-deployment optimization, and monitoring-process simulation—was proposed with the No. 42 turnout as the primary study subject and was implemented in actual monitoring operations. A MATLAB-based analysis and application program for turnout-zone wheel–rail forces was developed, enabling precise computation of force data. By integrating safety indices such as the coefficient of derailment and the rate of wheel load reduction, a comprehensive evaluation of the operational status of the No. 42 turnout was conducted. The principal findings are as follows:

1. Fine-scale modeling of critical turnout components was realized through the use of a parametric family library; the wheel–rail force monitoring scheme was optimized and integrated with 4D construction simulation, thereby effectively avoiding equipment installation clashes and refining construction workflow sequencing; the information fragmentation inherent in traditional two-dimensional drawings was resolved; technical handovers to monitoring personnel were conducted; construction efficiency and resource utilization were significantly enhanced; and alignment with green-construction principles was ensured.
2. A wheel–rail force analysis and application program for the turnout zone was developed based on MATLAB, through which systematic analysis from raw signal acquisition to safety evaluation was realized; the dynamic response characteristics at the turnout entrance, the circular-to-easement transition, and the easement-to-tangent transition were quantitatively elucidated, thereby providing technical support for turnout maintenance and design optimization.
3. Field measurements indicated that the rate of wheel load reduction at the circular-to-easement transition was comparatively greater, whereas lower rates were recorded at the turnout entrance and the easement-to-tangent transition. The maximum values of the coefficient of derailment—both inner and outer—at the turnout entrance, the circular-to-easement transition, and the easement-to-tangent transition were all found to lie below the limits prescribed by the national standard, thereby conforming to regulatory requirements. The safety and feasibility of the proposed scheme were thus validated, providing a theoretical basis for ensuring train operational safety.

Despite the demonstrated benefits, this study has certain limitations. The monitoring scheme was validated on the No. 42 turnout, which restricts the generalizability of the findings. To ensure broader applicability, future experiments should include a wider variety of turnout types and track conditions. Expanding the dataset to encompass extreme operating conditions and diverse traffic profiles will enhance the robustness and reliability of the analysis. Future work will focus on addressing these limitations by extending field deployments, integrating real-time analytics, and developing adaptive monitoring strategies tailored to a broader range of turnout geometries and service environments.

## Data Availability

The original contributions presented in this study are included in the article. Further inquiries can be directed to the corresponding author.
